# The effectiveness of generic emails versus a remote knowledge broker to integrate mood management into a smoking cessation programme in team-based primary care: a cluster randomised trial

**DOI:** 10.1186/s13012-021-01091-6

**Published:** 2021-03-20

**Authors:** Nadia Minian, Sheleza Ahad, Anna Ivanova, Scott Veldhuizen, Laurie Zawertailo, Arun Ravindran, Claire de Oliveira, Dolly Baliunas, Carol Mulder, Corneliu Bolbocean, Peter Selby

**Affiliations:** 1 Nicotine Dependence Service, Centre for Addiction and Mental Health, 1025 Queen St W, Toronto, ON M6J 1H4 Canada; 2 Department of Family and Community Medicine, University of Toronto, 500 University Ave, Toronto, ON M5G 1V7 Canada; 3 Campbell Family Mental Health Research Institute, Centre for Addiction and Mental Health, 250 College St., 1st floor, Toronto, ON M5T 1R8 Canada; 4 Institute of Medical Science, University of Toronto, Faculty of Medicine, 1 King’s College Circle, Medical Science Building, Toronto, ON M5S 1A8 Canada; 5 Department of Pharmacology and Toxicology, Faculty of Medicine, University of Toronto, Medical Sciences Building, Room 4207, 1 King’s College Circle, Toronto, ON M5S 1A8 Canada; 6 Centre for Addiction and Mental Health, 100 Stokes Street, Toronto, ON M5T 1P7 Canada; 7 Department of Psychiatry, University of Toronto, 250 College Street 8th floor, Toronto, ON M5T 1R8 Canada; 8 Centre for Addiction and Mental Health, 33 Russell Street, Toronto, ON M5S 2S1 Canada; 9 Institute of Health Policy, Management and Evaluation, University of Toronto, 155 College St, Suite 425, Toronto, ON M5T 3M6 Canada; 10 Centre for Health Economics and Hull York Medical School, University of York, Alcuin A Block, Heslington, York, YO10 5DD UK; 11 School of Public Health, The University of Queensland, Herston, QLD Australia; 12 Dalla Lana School of Public Health, University of Toronto, 155 College St Room 500, Toronto, ON M5T 3M7 Canada; 13 Queen’s University Department of Family Medicine, 220 Bagot St, Kingston, ON K7L 3G2 Canada; 14 Department of Preventive Medicine, University of Tennessee Health Science Center, 66 N. Pauline Street, Suite 633, Memphis, TN 3816 USA

**Keywords:** Remote knowledge broker, Smoking cessation, Mood management intervention, Knowledge translation strategies

## Abstract

**Background:**

Knowledge brokering is a knowledge translation approach that has been gaining popularity in Canada although the effectiveness is unknown. This study evaluated the effectiveness of generalised, exclusively email-based prompts versus a personalised remote knowledge broker for delivering evidence-based mood management interventions within an existing smoking cessation programme in primary care settings.

**Methods:**

The study design is a cluster randomised controlled trial of 123 Ontario Family Health Teams participating in the Smoking Treatment for Ontario Patients programme. They were randomly allocated 1:1 for healthcare providers to receive either: a remote knowledge broker offering tailored support via phone and email (group A), or a generalised monthly email focused on tobacco and depression treatment (group B), to encourage the implementation of an evidence-based mood management intervention to smokers presenting depressive symptoms. The primary outcome was participants’ acceptance of a self-help mood management resource. The secondary outcome was smoking abstinence at 6-month follow-up, measured by self-report of smoking abstinence for at least 7 previous days. The tertiary outcome was the costs of delivering each intervention arm, which, together with the effectiveness outcomes, were used to undertake a cost minimisation analysis.

**Results:**

Between February 2018 and January 2019, 7175 smokers were screened for depression and 2765 (39%) reported current/past depression. Among those who reported current/past depression, 29% (437/1486) and 27% (345/1277) of patients accepted the mood management resource in group A and group B, respectively. The adjusted generalised estimating equations showed that there was no significant difference between the two treatment groups in patients’ odds of accepting the mood management resource or in the patients’ odds of smoking abstinence at follow-up. The cost minimisation analysis showed that the email strategy was the least costly option.

**Conclusions:**

Most participants did not accept the resource regardless of remote knowledge broker strategy. In contexts with an existing KT infrastructure, decision-makers should consider an email strategy when making changes to a programme given its lower cost compared with other strategies. More research is required to improve remote knowledge broker strategies.

**Trial registration:**

ClinicalTrials.gov, NCT03130998. Registered April 18, 2017, (Archived on WebCite at www.webcitation.org/6ylyS6RTe)

**Supplementary Information:**

The online version contains supplementary material available at 10.1186/s13012-021-01091-6.

Contribution to the literature

- Implementation of remote knowledge brokers (rKB) to support integration of evidence-based treatment in primary care continues to grow, despite lack of evidence on how efficacious rKBs are. This study failed to demonstrate the superiority of a personalised rKB over generic emails. This is particularly relevant in the current situation of remote care provision and complete cessation of in-person KB activities due to the COVID-19 pandemic.
- This study provides decision-makers with relevant information to decide whether to use a rKB in systems with strong KT infrastructures including virtual components.
- Outcomes of this study also provide information related to the costs of KT strategies in general, something that is usually lacking in the published literature.

## Background

There is an increased call to use evidence-based practices (EBP) in the management and delivery of primary care [1, 2]. While funding agencies and policy- and decision-makers have promoted the application of EBP within primary care settings to enhance the quality of healthcare programmes and improve patient care, implementing new research into clinical practice, and sustaining these evidence-based interventions long term, is often challenging [3–5]. Various knowledge translation (KT) strategies have been used to help build capacity and encourage the implementation of EBP within healthcare settings, including training [6, 7], technology-enabled supports [8–10], financial incentives [5, 11], policy initiatives [5] and knowledge brokering [5, 12]. In Canada, the use of a knowledge broker (KB) is a common approach to bridge the gap between researchers and decision-makers [13, 14]. However, while KBs are well established within the private sector [1, 15, 16], evidence on their role and efficacy within healthcare settings has been largely anecdotal, and often inconclusive [12, 15, 17–20]. Given the costs and resources associated with traditional, in-person, models of knowledge brokering [20, 21], some programme implementers have shifted to remote KB (rKB) services, including virtual communities of practice (CoP), emails and phone calls [20, 22, 23]. However, the effectiveness of KBs operating from remote contexts has not been rigorously evaluated in primary care.

We conducted a cluster randomised controlled trial (RCT) to examine the effectiveness of two KT strategies in team-based multidisciplinary primary care settings (known as Family Health Teams [FHTs]) across Ontario, Canada, to increase healthcare provider (HCP) capacity in implementing an evidence-based mood management intervention within their existing smoking cessation programme. The intervention was operationalised through the Smoking Treatment for Ontario Patients (STOP) programme [24], an existing, in-person, smoking cessation treatment programme that partners with clinics across the province to provide up to 26 weeks of free nicotine replacement therapy (NRT) and behavioural counselling to treatment-seeking tobacco users. We chose this intervention as there is strong evidence demonstrating that integrating a psychosocial mood management component within smoking cessation programming can increase long-term quit rates among smokers with both current and past depression [25]. However, smokers with co-occurring depression are less likely to be treated for their tobacco use, often due to misconceptions regarding treatment approach and efficacy [26]. Thus, there was a need to develop an intervention to encourage HCPs to integrate mood interventions within their smoking cessation practice [26, 27]. Data from the STOP programme showed that 38% of FHT patients had current depression (determined by a score of 5 or higher on the Patient Health Questionnaire-9) or self-reported past depression, and these participants had significantly lower 6-month quit rates compared with patients without depression (33% vs. 40%, *p* < 0.001) [28]. This is consistent with the literature [25, 29, 30] which in addition shows that compared with the general population, individuals with depression are almost twice as likely to be smokers [31] and experience greater nicotine dependence, negative mood changes and higher rates of relapse when making a quit attempt [25, 29, 30].

The overall aim of this cluster randomised controlled trial (RCT) was to test a mid-range theory (a theory whose application is restricted to a certain subset of social phenomena relevant to a particular range of contexts [32]), where we hypothesised that a more intense and personalised intervention (rKB) would be more effective at enabling HCPs to provide their patients with mood management resources when needed, and ultimately help more smokers quit smoking, compared with a more passive intervention (generic monthly emails).

In this manuscript, we report on the three objectives set out in our trial protocol [33]:

1. To test the hypothesis that a personalised rKB (group A) would increase patients’ acceptance of a mood management resource relative to an active control condition of generalised email-based prompts (group B).
2. To test whether the personalised rKB also increased participants’ smoking quit rates at 6-month follow-up relative to the general email prompts.
3. To quantify the costs and benefits of the rKB (group A) relative to the general email prompts (group B).

A cluster RCT, with FHT clinics as the units of randomisation and STOP programme patients as the unit of analysis, was chosen to prevent contamination that would result if providers working within a clinic were exposed to both arms of the trial.

## Methods

To allow for replication of our study interventions, this trial adheres to reporting standards using the template for intervention description and replication (TIDieR) guide [34] and the CONSORT guidelines for cluster RCTs [35]. The completed TIDieR checklist is included as Additional File 1. The completed CONSORT checklist [35] for cluster RCTs is included as Additional File 2. The study methods described here are described in more detail in our protocol manuscript [33].

### Study design and setting

We conducted a pragmatic cluster RCT in FHTs (clusters) in Ontario implementing the STOP programme. FHTs joining the STOP programme were required to sign an Inter-Institutional Clinical Trial Collaborative Agreement, by which they consent to participate in the STOP study and conduct all STOP programme protocols in accordance with the agreement. In addition, HCPs delivering the STOP programme must receive training from a recognised education programme. Previous findings show that the majority of FHT HCPs implementing the STOP programme have attended training in an intensive tobacco cessation counselling programme, the Training Enhancement in Applied Counselling and Health (TEACH) Core course [36], while others identified being trained in less intensive programmes, including the Ottawa Model for Smoking Cessation [37], the Best Practice Champions [38] and the Quit Using and Inhaling Tobacco (QUIT) programme [24, 39]. The most common professional designations of HCPs implementing the STOP programme in FHTs are registered nurse (47.8%), pharmacist (19%) and nurse practitioner (12%). Other disciplines reported by STOP programme implementers include registered practical nurse, respiratory educator, social worker, addiction/mental health counsellors and health promoters.

As part of their role, HCPs are required to administer an initial baseline survey to treatment-seeking tobacco users who are interested in enrolling in the STOP programme. This survey includes questions about the patient’s current tobacco use, general health and sociodemographic information. HCPs are also responsible for providing patients with behavioural counselling and dispensing NRT during intake and at scheduled follow-up appointments. Additional resources and referrals to other FHT members can also be offered to patients based on any comorbid conditions and health behaviours reported.

## Implementation framework

This study was guided by the Interactive Systems Framework (ISF) for Dissemination and Implementation [40]. ISF outlines three interactive systems to implement scientific knowledge: the synthesis and translation system (“which distills information about innovations and translates it into user-friendly formats” [40]), the support system (“which provides training, technical assistance or other support to users in the field” [40]) and the delivery system (“which implements innovations in the world of practice” [40]). Each of these systems is described below.

### Pre-implementation

Prior to the launch of this trial, we examined the implementation climate of FHTs using a survey distributed to 125 STOP lead implementer(s) working in FHTs. The survey captured the three components of organisational readiness described by Scaccia et al [22]: motivation, general capacity and innovation-specific capacity. Motivation was defined as HCPs’ perceptions that the mood management intervention was compatible with the clinic values, was needed and would be useful to their patients. General Capacity was defined as the infrastructure, culture and context within the organisation in which the mood management intervention was going to be introduced. General capacities are associated with the ability to implement any innovation [41]. Innovation-Specific Capacity was defined as perceived knowledge, skills and abilities of HCPs to implement a mood management intervention. Based on answers to this survey, FHTs were grouped into two categories: most ready, and least ready. Given that completing the readiness survey was not a prerequisite to being randomised into the study, FHTs that did not complete the survey were classified together in a group labeled “unknown readiness”. For this trial, FHTs in Ontario, Canada, implementing the STOP programme, were the delivery system as outlined in the ISF.

After analysing the readiness survey, we invited FHTs to participate in two 60-min-long interactive webinars sharing best practices for integrating mood interventions into smoking cessation programming. These webinars formed the basis for the support system outlined in the ISF. Detailed answers from the readiness survey were used to develop the content of webinars which were delivered by the PI (PS). The recordings of these webinars can be accessed here: webinar 1: https://tinyurl.com/y9qhbee5, webinar 2: https://tinyurl.com/y8gmsfsb. The slide-decks of these webinars were part of the synthesis system outlined in the ISF.

### Trial design

FHTs (i.e. study clusters) were stratified by three levels of organisational readiness to implement a mood management intervention, and two levels of clinic size (estimated annual eligible patient enrollment), resulting in six strata. Within each of the six strata, a study co-investigator (DB) randomised clinics using a 1:1 allocation ratio to either group A (tailored rKB) or group B (monthly email prompts). The random assignment of treatment to FHT was computer generated using the ralloc command in Stata 14. All stratification and group allocation were performed prior to the initiation of the study. Participating FHTs were not informed of their allocation until the trial began. All authors, except SA who was the rKB and AI, SV and DB who conducted the analysis, remained blinded until the last follow-up survey was completed; AI, SV and DB were blinded until analysis of the primary outcome. Two study staff were un-blinded to allocation results so as to facilitate implementation of the random allocation sequence within the rKB or email groups. Additional details about determination of the readiness and size strata as well as the randomisation process can be found in our protocol manuscript [33].

### Eligibility criteria

#### Cluster (FHT) level

Ontario FHTs who were implementing the STOP programme at the time of randomisation in February 2018, and used the STOP portal for programme operations, including patient enrollment, were eligible to participate in the trial.

#### Patient level

Patients who provided consent to participate in the STOP programme, and enrolled in person at an eligible FHT with their baseline enrollment survey completed in English by a HCP, using the STOP portal in real-time, were eligible to participate in the study. Patients who completed their baseline enrollment on paper, or in French, were excluded from the trial. In order to be eligible to receive the mood management intervention at the time of enrollment, patients must either have reported a past diagnosis of depression or have scored 5 or higher on the Patient Health Questionnaire (PHQ-9) a validated and widely-used screen for major depressive disorder, which was already part of the baseline assessment package in all FHTs participating in this study [42].

### Interventions

#### Mood management intervention

**Fig. 1:**
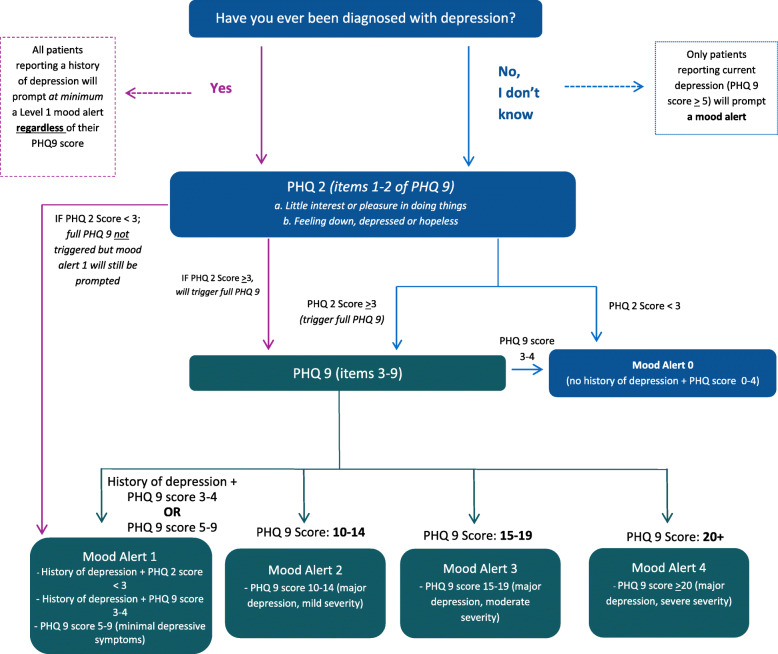
STOP Portal levels of depression severity

Based on their PHQ-9 score, which was automatically calculated by the online portal prior to survey completion, patients were grouped into one of four possible levels of depression severity: (1) minimal depressive symptoms (PHQ-9 score < 4 and a reported history of depression, or PHQ-9 score 5-9); (2) major depression with mild severity (PHQ-9 score 10–14); (3) major depression with moderate severity (PHQ-9 score 15–19); or (4) major depression with severe severity (PHQ-9 score 20 or greater) (see Fig. 1). For this study, and as part of the synthesis and support systems of the ISF framework, we embedded a computer decision support system into the STOP portal in order to guide all HCPs with delivering a mood management intervention to patients. The intervention included a tailored brief intervention, based on the patient’s level of depression severity and Canadian Network for Mood and Anxiety Treatments guidelines [43], and a self-help educational resource on mood management and smoking cessation (Additional file 3). The latter was adapted from the work of Munoz and colleagues [44]. The computerised alerts and online enrollment surveys were the same for patients in either intervention arm.

#### Treatment arms

The lead STOP programme implementer at each FHT allocated to group A received personalised phone and email-based support from the rKB, in order to help HCPs build capacity and encourage implementation of the mood management intervention. This was conceptualised under the support system of the ISF. Within group B, lead STOP programme implementers at each FHT received a generalised monthly email containing a PDF resource with information on treating smokers with mood disorders, which were part of the ISF synthesis system. Topics included how to provide a brief mood intervention, working with patients with co-morbid conditions and managing suicidal ideation (see Additional file 4 for the first generalised email we sent out). Some FHTs operated multiple clinics; in these cases, the lead implementer at each clinic received the intervention.

The rKB held a Master’s of Science specialising in research and had prior experience working in an addictions and mental health setting. The frequency of communication between each FHT and the rKB, and the content discussed, depended on the individual needs of each clinic/lead implementer. For more information describing the role of the rKB implemented in this study, please refer to our manuscript (Minian N, Ahad S, Zawertailo L, Ravindran A, de Oliveira C, Baliunas D, et al.: Conceptualizing the role of a remote knowledge broker: a case study in primary care settings, in preparation).

### Outcomes

The study had three outcomes. The primary outcome was acceptance of the mood management resource by eligible patients. This dichotomous outcome was collected via the STOP online portal and measured as positive if the HCP responded “Patient accepted the resource” to the question “Did the patient accept or decline the mood resource?” If the HCP indicated that the “Patient declined the resource” or the HCP responded “No” to the automated prompt, “Please provide this patient with a resource on mood management”, the primary outcome was negative, and interpreted as “patient did not accept a mood management resource”. In 18 cases (*n* = 8 in group A and *n* = 10 in group B), either the online portal failed to activate the mood intervention pathway despite the patient being eligible for a mood resource or the system failed to record the HCPs’ response to the mood management resource provision or patient acceptance questions. In those cases, the primary outcome was coded as negative.

The secondary outcome was patient smoking abstinence at 6-month follow-up. Six months after enrollment into the STOP programme, patients were asked to complete a follow-up survey regarding their smoking status, which was administered via phone by trained study staff, via email using a survey link, or by HCPs during a visit to the FHT. Patients had one month from their 6-month enrollment anniversary before the survey expired. Abstinence from smoking was defined as a negative response to the seven-day point prevalence question, “Have you had a cigarette, even a puff, in the last 7 days?” Using a seven-day window to calculate point prevalence abstinence from smoking is the most common time frame researchers’ use [45]. In addition, the validity of self-reported abstinence from smoking has been shown to be a good estimate of smoking status [46].

The tertiary outcome was the costs of delivering each intervention arm, which, together with the effectiveness outcomes, were used to undertake an economic evaluation. In turn, the objective of the economic evaluation was to undertake a comparative assessment of the associated costs and benefits related to delivering each intervention arm (i.e. the tailored rKB arm and the generic email arm).

### Sample size

Previous STOP programme enrollment was used to predict eligible FHTs and expected clinic enrollment to perform randomisation allocation. The study was powered to detect an absolute risk difference of 0.06 with alpha = 0.05 and power = 0.80. Sample size calculations took into account the intra-cluster correlation (ICC) within FHT clinics and variation in FHT sizes [47]. Previous work with HCPs being prompted to deliver a self-help resource using the STOP portal provided an expected ICC of *ρ* = 0.032, an average annual enrollment of 24 patients per clinic, and cluster size coefficient of variation (CV) of 1.24 [48]. This yielded a sample size estimate of 2448 patients (1224 per arm).

### Statistical analysis

Descriptive statistics were generated for patient and FHT clinic level characteristics for each of the two treatment arms. Patient characteristics were measured at enrollment, while FHT level characteristics were obtained from STOP programme administrative data. Generalised estimating equations (GEE) using a population-averaged method, with an exchangeable correlation matrix and robust standard errors, were used to examine the association between treatment groups on the primary and secondary outcomes and to account for clustering. The study design stratification variables (organisational readiness and size) were included as covariates in the model. Other covariates were: age, gender, employment status, education level, household income, smoking status, willingness to quit smoking in the next 30 days, self-reported First Nations, Inuit or Métis (FNIM) status, past year alcohol use, past 30-day marijuana use, past 30-day opioid use, total PHQ score (the sum of completed items) and self-reported lifetime history of depression, anxiety, schizophrenia, bipolar disorder, substance use disorder, alcohol use disorder and problem gambling. The same set of covariates was used for both the primary and secondary outcome models. All covariates are measures of constructs specified in the study protocol, with the exceptions of problem gambling and FNIM status. Problem gambling was added in order to more completely capture psychiatric morbidity, and FNIM status because of the unique health challenges faced by this population [49].

The study protocol specified a sensitivity analysis to determine whether multiple imputation should be performed. However, due to the amount of missing data for some baseline covariates (Table 1), multiple imputation was used, without a previous sensitivity analysis, for both models [50].

**Table 1:** Baseline patient and FHT characteristics for main analytic sample (*n* = 2763)

	Group A (knowledge broker)	Group B (monthly emails)	Total missing
**Patient level**	*n* = 1486	*n* = 1277	*n* (%)
Age in years (mean, SD)	51.1 (13.5)	50.4 (13.7)	0 (0)
Male	580 (39)	473 (37)	0 (0)
First Nations, Inuit or Métis	70 (5)	116 (9)	50 (2)
Graduated high school	722 (50)	564 (49)	161 (6)
Currently employed	533 (36)	483 (38)	28 (1)
Household income above 40k	309 (37)	277 (40)	1220 (44)
Daily smoker	1398 (94)	1191 (93)	1 (0)
Willing to set a quit date in next 30 days	1073 (84)	851 (80)	421 (15)
PHQ9 (mean, SD)	4.9 (7.0)	4.2 (6.7)	0 (0)
Consumed alcohol in past year	976 (66)	823 (65)	27 (1)
Marijuana use in past 30 days	520 (35)	443 (35)	27 (1)
Opioid use in past 30 days	376 (26)	305 (24)	31 (1)
Lifetime history of depression^a^	1396 (94)	1205 (95)	11 (0)
Lifetime history of anxiety^a^	1038 (71)	873 (69)	35 (1)
Lifetime history of schizophrenia^a^	47 (3)	35 (3)	44 (2)
Lifetime history of bipolar disorder^a^	140 (10)	99 (8)	52 (2)
Lifetime history of substance use disorder^a^	187 (13)	112 (9)	48 (2)
Lifetime history of alcohol use disorder^a^	192 (13)	138 (11)	45 (2)
Lifetime history of problem gambling^a^	36 (2)	26 (2)	43 (2)
**Cluster (FHT) level**	**(*n*** **= 58)**	**(*n*** **= 53)**	
Patient Participants per cluster (mean, sd)	25.6 (36.9)	24.1 (18.2)	
Year clinic enrolled first patient in the STOP programme			
2011	36 (62)	29 (55)	
2012	11 (19)	10 (19)	
2013	4 (7)	3 (6)	
2014	5 (9)	3 (6)	
2015	2 (3)	5 (9)	
2016	0 (0)	3 (6)	
2017	0 (0)	0 (0)	
2018	0 (0)	0 (0)	
Local Health Integration Networks^b^ (health regions in Ontario)			
Central	2 (3)	5 (9)	
Central East	5 (9)	3 (6)	
Central West	2 (3)	1 (2)	
Champlain	6 (10)	5 (9)	
Erie-St.Clair	6 (10)	4 (8)	
Hamilton Niagara Haldimand Brant	3 (5)	5 (9)	
Mississauga Halton	3 (5)	0 (0)	
North East	6 (10)	10 (19)	
North Simcoe Muskoka	1 (2)	3 (6)	
North West	4 (7)	5 (9)	
South East	7 (12)	3 (6)	
South West	7 (12)	3 (6)	
Toronto Central	1 (2)	3 (6)	
Waterloo Wellington	5 (9)	3 (6)	

Values are numbers (percentages of non-missing) unless stated otherwise. *SD =* standard deviation

^a^Self-reported lifetime history of past diagnosis

^b^Local Health Integration Networks (LHINs) are agencies established by the Government of Ontario to plan, coordinate, integrate and fund health services at a local level. They represent health regions across the province. A total of fourteen LHINs have been established across Ontario

The missingness models included all the variables from the main analyses, as well as the number of clinical visits within the first 6 months of enrollment, the total amount of NRT supplied at these visits (in weeks), average cigarettes smoked per day at baseline, time to first cigarette after waking (within 5 min, 6–30 min, 31–60 min, more than 60 min), number of past lifetime quit attempts (0, 1–5, 6–10, 11+) and smoking status at other programme follow-ups and clinical assessments not included in the present study (follow-ups at 3 months and 12 months post-enrollment, and whether abstinence was recorded at any clinical visit). A single missing value for the smoking status variable was also set to “daily” (the value in 94% of cases) to ensure convergence of some missingness models. Using Stata 16’s MI procedures, 20 imputed datasets were generated, the substantive models were fit using each and results combined using Rubin’s rules. All analyses were conducted using Stata v14 and v16 [51].

### Economic evaluation

A comparative assessment of the associated costs and benefits (as defined by outcomes 1 and 2) related to delivering each arm of the intervention was conducted via an economic evaluation from the perspective of the public third party payer (i.e. the Ontario healthcare system), in line with the guidelines of the Canadian Agency for Drugs and Technologies in Health [52]. We accounted for all relevant costs associated with delivering each arm of the trial. Intervention costs included the costs of developing, maintaining and running each arm, costs of personnel and training and costs of supplies and services, among other things. We used the average hourly wage rate (including benefits) for each staff member involved to obtain the cost of their time allocated to the intervention. Other costs, such as costs of supplies and services related to the delivery of the intervention (telecommunications, printing, etc.), were obtained from institutional expense records. All costs were expressed in 2018 Canadian dollars.

### Ethics approval

This study was approved by the Research Ethics Board at the Centre for Addiction and Mental Health (protocol number 065-2016) as well as registered on ClinicalTrials.gov (ID: NCT03130998).

## Results

### Pre-intervention-readiness survey

The readiness survey was shared with all FHTs who were actively participating in the STOP programme and had a lead implementer in place at the time the survey was sent out (*n* = 125). Eighty-four FHTs completed the readiness survey (67% response rate). Results showed that 68% of providers were motivated to implement a mood management intervention as part of smoking cessation programming in their FHT clinic (score of 5 or higher; mean 5.38, SD 1.81); 63% reported their organisation had the general capacity to implement a mood management intervention (mean 5.28, SD 1.67); but only 31% believed that their organisation had the specific capacity to do so (mean 3.85; SD 1.96).

FHTs were grouped into two categories: most ready (high readiness; *n* = 44), and least ready (low readiness; *n* = 40). Given that responding to the readiness survey was not an eligibility criteria for participation in the trial, FHTs who were eligible to participate in this trial but did not answer the questionnaire (*n* = 39) were classified together in a group labeled “unknown readiness”.

### Intervention

**Fig. 2:**
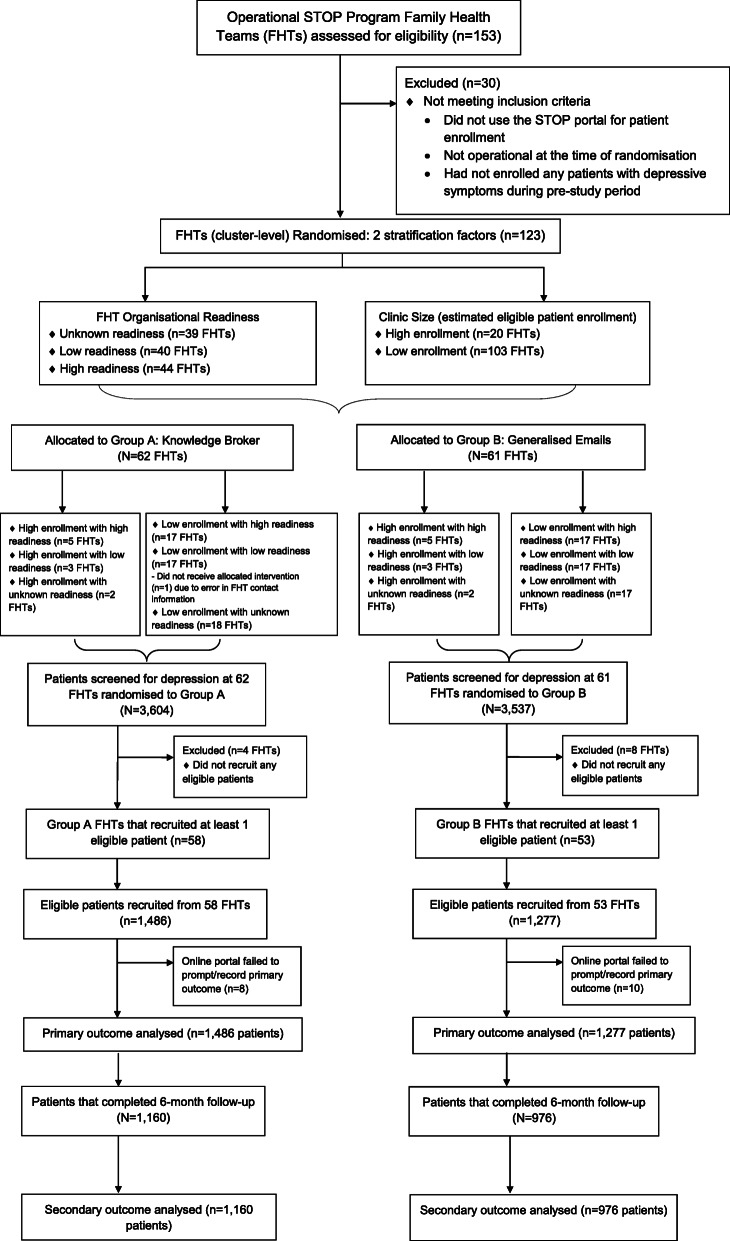
CONSORT flow diagram

At the time of randomisation, 153 FHTs were participating in the STOP programme and assessed for eligibility (28 of these FHTS had not been shared the readiness survey since they did not have an active STOP implementer at the time or joined the STOP programme after the survey was sent out). These clinics had enrolled at least one patient, in English, with valid consent, during the pre-study period. Additional eligibility criteria were applied to this sample, including the clinic being operational at the time of randomisation, and using the STOP portal during the pre-study period and enrolling at least one patient with depressive symptoms. This resulted in 123 FHTs being randomised into the trial. Sixty-two FHTs were randomised to group A (rKB) and 61 FHTs were randomised to group B (generalised emails). Fifty-eight FHTs from group A and 53 FHTs from group B enrolled at least one eligible patient into the study. Figure 2 shows our CONSORT flow diagram, including the number of FHTs enrolled, allocated to each intervention and included in our primary and secondary data analyses. Table 1 shows the number and types of practices who were enrolled, allocated and analysed in the study. The study sample included 2763 eligible patients; *n* = 1486 from group A and *n* = 1277 from group B. The observed ICC was *ρ* = 0.14, and the average enrollment was 25 patients per clinic across 111 FHTs.

There were minor differences in self-identification as First Nations, Inuit or Métis, high school completion, willingness to set a quit date in the next 30 days, and lifetime history of substance use disorder. There were few notable differences between FHTs, with the exception of FHTs in group A having started implementing the STOP programme slightly earlier and having less representation from the northern areas of Ontario. The FHT characteristic and the patient demographics, separated by treatment group, are presented in Table 1.

Between February 2018 and January 2019, 7175 patients were screened for depression and 2765 (39%) reported current and/or past depression. The primary outcome is presented in Table 2. Overall, 29% (437/1486) and 27% (345/1277) of patients accepted the mood management resource in group A and group B, respectively. The adjusted GEE showed that there was no significant difference between the two treatment groups in the odds of eligible patients receiving the mood management resource.

**Table 2:** Adjusted odds ratio and 95% confidence intervals for the primary and secondary outcomes

Outcomes^a^	No. (%) in group A (knowledge broker)	No. (%) in group B (monthly emails)	Intra-cluster correlation coefficient^b^	Adjusted odds ratio^c^ (95% CI)	*P* value
Primary: Patient accepted the mood resource at enrollment (*n* = 2763)	437/1486 (29)	345/1277 (27)	0.141	0.93 (0.60, 1.43)	0.73
Secondary: Patient quit smoking at 6-month follow-up (*n* = 2136)	345/1160 (30)	279/976 (29)	0.010	1.11 (0.91, 1.35)	0.32

^a^The primary outcome was derived from healthcare providers’ response to the online STOP portal prompt at patient enrollment. The secondary outcome was measured at patients’ 6-month follow-up. The secondary outcome model was limited to patients who responded to the 6-month outcome survey

^b^Based on unadjusted models

^c^Both models were adjusted for study stratification variables (organisational readiness and size) and the following patient-level variables measured at enrollment: age; gender; self-reported First Nations, Inuit or Métis status; employment status; education level; household income; smoking status; willingness to quit smoking in the next 30 days; past year alcohol use; past 30-day marijuana use; past 30-day opioid use; sum PHQ-9 score and self-reported lifetime history of depression, anxiety, schizophrenia, bipolar disorder, substance use disorder, alcohol use disorder and problem gambling

The secondary outcome is also presented in Table 2. The rate of response to the 6-month follow-up survey was 77% (2136/2763 eligible patients completed the survey between August 2018 and August 2019). The remaining participants did not respond to repeated contact attempts by email and phone. The response rate was similar in both groups (group A, 1160/1486, 78.1%; group B, 972/1277, 76.1%; *χ*^2^ = 1.48, p=0.22). The crude quit rate from smoking cigarettes at follow-up was 29.7% (345/1160 patients) in group A and 28.5% (279/976 patients) in group B. Twenty-three percent of patients did not complete the 6-month follow-up survey and were therefore missing the secondary outcome. After MI, these proportions were 27.8% (95% CI = 25.4–30.2%) in group A and 27.5% (95% CI = 24.8–30.3%) in group B. The adjusted GEE showed that there was no significant difference between the treatment groups in the patients’ odds of smoking abstinence at follow-up.

Finally, the tertiary outcome, which was used in the economic evaluation, is presented in Table 3. Given that there was no difference in outcomes between arms, undertaking a cost-effectiveness analysis was no longer feasible. Instead, we conducted a cost-minimisation analysis, which compares the costs between two interventions with equivalent outcomes. The costs of delivering the tailored rKB (group A) and the generalised email (group B) arms were categorised into costs, which were specific to each arm and those common to both arms. The costs of delivering the tailored rKB included training the rKB, preparing study instruments, communicating with HCPs of each clinic and preparing FHT-specific data to share with HCPs ($11,839.81), while the costs of delivering the generalised emails included costs with the preparation of resources, communicating with FHTs and training research staff and students ($10,611.17). Costs common to both arms ($25,744.25) included costs associated with meetings with co-investigators and vendors to discuss the study design and implementation; developing study instruments for data collection, analysis and evaluation of outcomes; preparing screening tools and treatment guidelines associated with delivering mood interventions; disseminating an online webinar to FHTs to increase their capacity in delivering the intervention; communications to funders, stakeholders and study participants; and developing and analysing a readiness survey distributed to FHTs before and after the initiative to assess the organisational readiness to implement the mood intervention in practice. Overall, our analysis suggests that the generalised email arm is the cost-minimizing arm, costing $1228.65 less than the tailored rKB arm (Table 3).

**Table 3:** Cost minimisation analysis

	Generic email arm^a^ (A)	Tailored rKB arm^b^ (B)	Both arms (C)	Generic email arm total (A) + (C)	Tailored rKB arm total (B) + (C)	Difference [(B) + (C)] − [(A) + (C)]
Intervention costs^c^	10,611.17	11,839.81^d^	25,744.25^e^	36,355.42	37,584.06	1228.65
Literature review and intervention preparation costs	–	–	5779.33	5779.33	5779.33	0
Suicidal ideation protocol costs	–	–	3953.87	3953.87	3953.87	0
**Total costs**	10,611.17	11,839.81	35,477.45	46,088.62	47,317.26	1228.65

^a^Participants in the generic email arm received monthly messages (related to smoking and depression) exclusively via email

^b^Participants in the rKB arm received personalised support through phone and email-based check-ins

^c^Intervention costs for the generic email arm included costs with the preparation of resources, communicating with FHTs and training research staff and students; Intervention costs for the tailored rKB arm included training the rKB, preparing study instruments, communicating with HCPs of each clinic and preparing FHT-specific data to share with HCPs

^d^This value includes delivery-related costs (not intervention costs) of the tailored remote knowledge broker arm (11,819.81) and the cost of telecommunications (emails, phone calls) (20.00)

^e^This value includes the cost of delivering both arms (22,167.25), the cost of running two webinars (1650.00) and the cost of mailing materials to participating Family Health Teams (1927.00)

For the cost-minimisation analysis, we conducted a sensitivity analysis that included the costs we encountered in the study that are not necessarily required to implement the intervention, but that may be undertaken if additional work is required to tailor the intervention in other settings or jurisdictions. Specifically, in this sensitivity analysis, we included the costs associated with:

1. The development of the study’s protocol which required undertaking literature reviews to determine the best available evidence in the field and conducting a readiness survey to assess FHTs’ readiness to adopt a mood management intervention as part of the STOP programme.
2. The development of a suicide risk assessment protocol for non-clinical research staff, which was implemented 6 months after the initiation of the trial to examine long-term changes in depression severity (measured via PHQ-9 score) among patients enrolled in the STOP programme. Thus, we also included the cost associated with undertaking literature reviews as well as the cost of implementing the suicidal ideation protocol in a sensitivity analysis.

## Discussion

For this study, we tested a mid-range theory, where we hypothesised that a more intense and personalised intervention (rKB) would be more effective at enabling HCPs to provide their patients with mood management resources when needed, and ultimately help more smokers quit smoking, compared with a more passive intervention (generic monthly emails). The results of this adequately powered study show that our mid-range theory was not supported; we failed to detect a statistically significant difference between a personalised rKB and a generic email-based intervention at facilitating the delivery of a mood management intervention into an existing smoking cessation programme within primary care settings (namely FHTs). The results of this trial also failed to detect a significant difference between a personalised rKB and a generic email-based intervention on patient smoking cessation at 6-month follow-up. The cost minimisation analysis showed that an email intervention is less costly of these two KT strategies. These results need to be understood within the context in which they took place. Prior to implementing the mood management intervention, the STOP programme already had a strong infrastructure that incorporated many virtual KT components, including online continuing education courses available through the Training Enhancement in Applied Counselling and Health Project [36], an active Listserv, and a CoP with bimonthly meetings for HCPs to learn and exchange new information related to tobacco addiction treatment. These strategies are well known to improve knowledge and clinical practice behaviours [53] as they allow HCPs to mutually engage in processes such as *de-centralised decision-making* and *thinking together* [54]. In addition to the existing KT infrastructure, for this trial, we also offered two webinars to train HCPs and embedded a computer decision support system to guide all HCPs with delivering a mood management intervention to patients with current and past mood disorders. Although the rKB offered both knowledge and tailored support beyond that of a CoP, it is possible that the existing KT resources available to STOP implementers, including the integration of a decision support system, were already providing some of the benefits of a KB. Thus the addition of the rKB may have led to an oversaturation of information for HCPs [55], hence revealing no statistically significant difference. Therefore, in settings where there is a strong KT infrastructure the added cost of a rKB might not be justified. In this study, less than 30% of patients who could benefit from a mood management intervention received it, highlighting the need for effective implementation strategies and a theoretical understanding of how to increase the adoption of a mood management intervention. Given that our pre-implementation results, which were based on Scaccia et al.’s R = MC^2^ theory [56], showed that most HCPs were motivated to implement a mood management intervention but needed help with specific capacity, we might want to explore cognitive theories that can influence the adoption of EBPs. One psychological theory that could be explored further is the parallel dual processing models of reasoning [57, 58] which suggests that two cognitive modes of information processing are in constant operation as humans reason; one is a fast, experiential mode and the other one is a rational conscious mode [57, 58]. The rKB and emails may have influenced the more rational, conscious mode, but offered little for the experiential mode. Finding implementation strategies that influence both might be an important way to facilitate the uptake of evidence into practice.

Our results differ from previous studies, which found that KBs were effective at enhancing HCP capacity [59] and improving practice change, compared with the passive dissemination of hardcopy and electronic instructions [60], and were also successful in facilitating the implementation of EBPs [61]. However, these studies were based on face-to-face meetings with stakeholders [59–61], rather than remote methods of communication reported in our study. This lack of in-person meetings may have, in part, contributed to the differences observed from earlier research. Previous authors exploring technology-based KT strategies in healthcare have reported challenges, including lack of engagement and low prioritisation by end users [18, 20, 22, 62]. It is possible that the success of KB interventions, beyond that of email-based interventions, require at least an initial face-to-face interaction in order to establish a meaningful connection and thoroughly explain the initiative, before shifting to remote methods of brokering [20].

Despite this notion, findings from our trial are comparable with results from an RCT conducted by Dobbins et al., who found that an in-person KB was not more effective than tailored messaging, for promoting evidence-informed decision-making in public health [63]. Similar to the authors’ remarks, we consider that KB success may be influenced by the prioritisation of research evidence within an organisation, whereby stakeholders with low perceived research culture and priorities may benefit from a KB more than those with high research culture [63]. We also consider that within the context of FHTs delivering smoking cessation treatment, simple KT interventions may be just as effective as more complex, multicomponent KT strategies [63, 64]. Although the email intervention (group B) was generalised across FHTs, and less personalised than the rKB, both strategies contained relevant and accessible information for the HCP, which are important for facilitating practice change [63]. Given that many clinicians working in primary care are often faced with competing priorities and limited time, the monthly email resources may have provided just the right amount of digestible information, which HCPs could review on their own time, rather than having to dedicate time toward formal phone check-ins with the rKB. In addition, more in-depth tools and resources shared by the rKB may have been too rigorous for HCPs working within an interdisciplinary environment, whereby more intensive interventions would be offered by mental health specialists.

The evidence we provided related to costs and outcomes associated with mood management interventions within smoking cessation programming demonstrates that the generalised email arm is the cost-minimizing arm compared with the tailored rKB arm. Given that once email content is prepared there are no costs of scaling it up, whereas offering support from a rKB does incur more costs as additional clinics are included, implementing an email-based KT intervention may be more feasible to integrate within interdisciplinary primary care organisations. This result might inform future policy decisions regarding the cost-effectiveness of mood management interventions within single-payer healthcare systems.

### Strengths and limitations

One of the main strengths of our trial was the pragmatic design testing the real-world effectiveness of the rKB intervention, and the large sample size utilised, which included 123 FHTs across Ontario and 2763 patients. Conducting an implementation readiness assessment also allowed us to tailor our KT materials (webinars, emails and rKB) to the needs of HCPs in order to increase uptake in both groups. In addition, by stratifying FHTs based on implementation readiness, we were able to account for the differences in organisational readiness between both groups.

A limitation to our study design was the lack of a control arm (i.e. no intervention at all), which would have provided an additional comparison to assess the effectiveness of both the rKB and the general emails for providing implementation support to HCPs. However, this was not the planned purpose of the trial and would have required a larger sample size. In addition, given the evidence supporting the integration of mood interventions within smoking cessation programming [25], and results from our readiness survey, where only 31% of HCPs reported having the specific capacity to implement mood management treatment, we felt it was important to provide all FHTs with some form of intervention support, varying in intensity, rather than no intervention at all. Finally, few programmes would introduce an automated treatment pathway with no support or training whatsoever, and including this as the control condition might therefore provide a somewhat artificial comparison.

A second limitation was that our primary outcome measure did not provide a full picture of how the rKB versus the generalised emails may have impacted HCP decision-making over the intervention period. For instance, the rKB may have improved HCPs’ knowledge and skills in delivering mood interventions within smoking cessation treatment and influenced the implementation of FHT policies related to mood management. However, while these are important outcomes, in order for the mood management intervention to work, smokers with current or past depression must *accept* the intervention; thus, we chose this as our primary outcome.

Although HCPs were assigned to two different treatment groups, we did not account for whether HCPs in group A were reached by the rKB and did not ascertain whether HCPs in group B actually read their monthly emails. However, given that the purpose of this study was to implement and examine mood management interventions in a real-life pragmatic treatment programme, our outcomes are likely more generalizable to real-world treatment settings where HCPs may be busy and not necessarily responsive to the communications they receive. Our secondary outcome was also not available for the 23% of patients who did not complete a 6-month follow-up. Another limitation is that patients who completed their baseline enrollment on paper, or in French, were excluded from the trial. Although there is no reason to think that their response to the intervention would have differed from those of included patients, their removal reduces the representativeness of the final sample. There is also the possibility of contamination of knowledge; HCPs working in FHTs assigned to group A might share some of the KB insights with HCPs from group B, and similarly, HCPs from group B might forward emails with HCPs working in FHTs assigned to group A. This contamination could have potentially compromised the effect of the trial, leading to a more conservative reporting estimate of the study’s overall effect. To our knowledge, however, as detected during a rKB phone call, only one HCP was exposed to both arms of the trial, that is they were employed at both a FHT assigned to group A and a FHT assigned to group B. Further, the occurrence of HCPs concurrently working at two STOP FHTs is low, and unlikely to have an impact on our results.

## Conclusions

This large study contributes to the implementation science literature by empirically testing a mid-range implementation theory (that active implementation strategies are more effective than passive ones) and showing that in the particular context it was tested, this theory was inaccurate. In addition, the results of this study show that the passive strategy is less costly to implement and sustain over the long term. More research is needed to examine in which contexts (e.g. sites without an existing KT infrastructure) active implementation strategies are more effective than passive ones. The study also provides a real-world example of how the Interactive Systems Framework for Dissemination and Implementation can be used in practice to guide implementation.

Future research could examine if dosage, number of interactions and /or total time spent, between the rKB and HCPs was a contributing factor in the success of the intervention. Patient involvement in requesting the intervention should also be studied to increase the overall implementation of this evidence-based practice in primary care settings. Finally, future work will also examine if HCPs continue to offer the mood intervention to patients despite the cessation of the rKB and the emails.

## Supplementary Information

**Additional file 1.** TIDieR checklist.

**Additional file 2.** CONSORT checklist.

**Additional file 3 **Mood Management Resource – *Self-awareness: managing your mood.* Description of data: A self-management resource offered to patients as part of the mood management intervention.

**Additional file 4.** Sample of generalized monthly email. Description of data: Sample of the first generalized monthly email that was sent to the lead implementer(s) at FHTs allocated to Group B.

## Data Availability

The datasets generated and/or analysed during the current study are not publicly available due to the fact that they contain personal health information, but are available from the corresponding author on reasonable request.

## Abbreviations

AFHTO: Association of Family Health Teams of Ontario
CAMH: Centre for Addiction and Mental Health
CIHR: Canadian Institute for Health Research
CoP: Community of Practice
EBP: Evidence-based practice
FHT: Family Health Team
GEE: Generalised estimating equations
HCP: Healthcare provider
KB: Knowledge broker
KT: Knowledge translation
NRT: Nicotine replacement therapy
PHQ-9: Patient Health Questionnaire
RCT: Randomised controlled trial
rKB: Remote knowledge broker
STOP: Smoking Treatment for Ontario Patients
TEACH: Training Enhancement in Applied Counselling and Health
