# Supplementary material for: The effectiveness of generic emails versus a remote knowledge broker to integrate mood management into a smoking cessation programme in team-based primary care: a cluster randomised trial
Source: Implement Sci. 2021 Mar 20;16:30. doi: 10.1186/s13012-021-01091-6 (PMC7980670; doi:10.1186/s13012-021-01091-6)
Supplement: Supplementary file 3 — Additional file 3 Mood Management Resource – Self-awareness: managing your mood. Description of data: A self-management resource offered to patients as part of the mood management intervention. [file 13012_2021_1091_MOESM3_ESM.pdf]

Self-awareness

Managing Your Mood

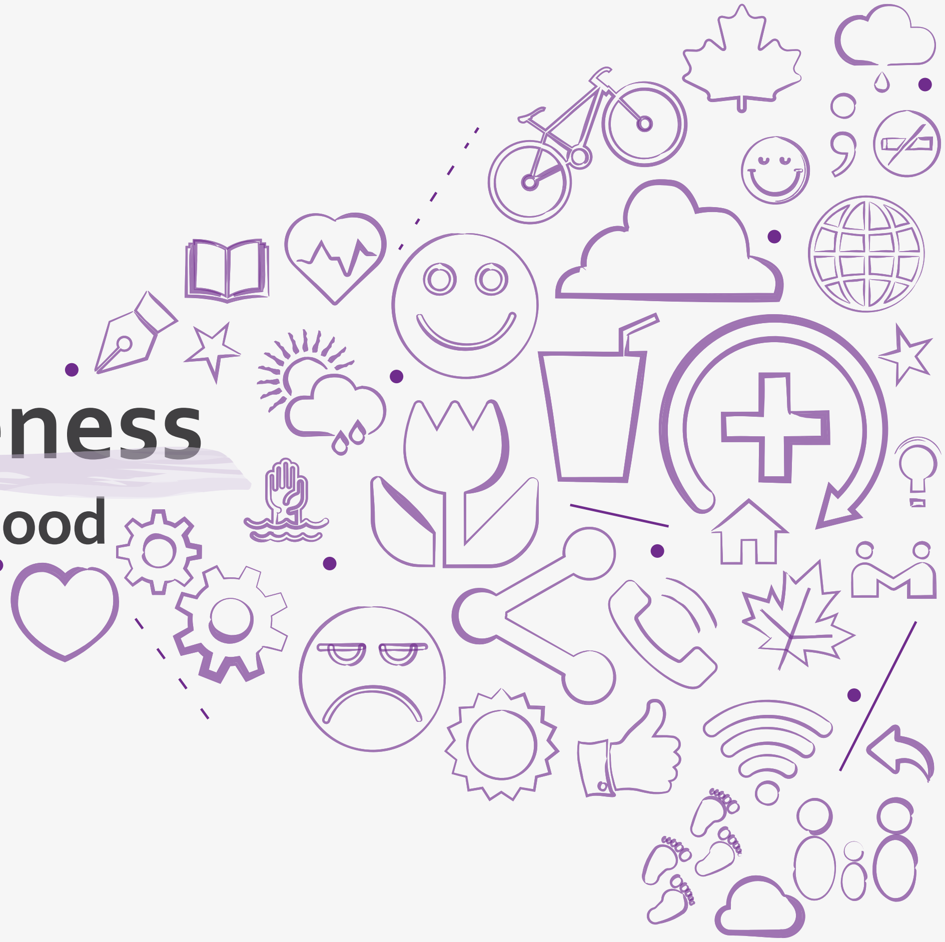

camh NDS
Nicotine Dependence Service

Welcome to the Self-Awareness Workbook for managing your mood! The workbook offers guidance, tips, tools, and resources to help manage depressive symptoms while trying to quit or reduce smoking.

Why is this workbook important?

People trying to quit smoking can feel low mood and experience withdrawal symptoms, which can often feel like depressive symptoms. These depressive symptoms can make it harder to quit smoking and can also lower your confidence in your ability to successfully quit.

Resources and strategies to manage mood, such as those found in this workbook, can help increase success in quitting smoking. This workbook offers activities and guidance to help you cut down or quit smoking while trying to manage negative feelings that might come up during your journey.

There is no right or wrong way to use this workbook. You can take your time working through the activities and repeat them as often as you need. By working through the activities in this workbook, you can better understand the connection between quitting smoking and changes in your mood. You can also be more ready for any feelings that may come up – both positive and negative – while you work to quit or reduce smoking.

Thank you for including us in your journey. You can make the change, and we are here to help!

Key features of this workbook:

Internal and external supports....................3

Understanding your current mood....................4

Activities to help you manage your mood and reduce smoking....................5

Strategies for maintaining a healthy mood....................11

Internal and external supports

It can feel overwhelming when you are trying to make changes to your behaviours, which can make you feel depressed or anxious. Getting support from others including family, friends, and professionals can help you with making the changes you desire.

Below is a list of internal and external supports that may help you manage any changes in emotions you experience as you reduce or quit smoking, or work to improve your mood. See your health care provider or a mental health professional if your symptoms don't go away or get worse.

External supports

The following are a few resources to help you find support near you:

ConnexOntario provides free and confidential health services information for people experiencing problems with alcohol and drugs, mental illness or gambling: www.connexontario.ca

Ontario Mental Health Helpline provides information about mental health services in Ontario
Call 1-866-531-2600 or visit their website: http://www.mentalhealthhelpline.ca/

Distress and Crisis Ontario offers support and a variety of services to people looking for someone to talk to when feeling lonely or down, or having suicidal thoughts. Find your closest Distress Centre at: http://www.dcontario.org/

Smokers' Helpline is a free, confidential service offering support and information about quitting smoking and tobacco use. There are 4 ways to get help from Smokers' Helpline:

1. Call +1 (877) 513 - 5333 or visit www.smokershelpline.ca
2. Join the online quit program for tips, tools and support 24/7
3. Sign up for customized text messages offering support
4. Access free booklets on smoking and quitting

Internal Supports

You can also seek help from the people in your life both personal and professional. Make a list of people that can support you while you quit or reduce smoking, including support groups.

Who are some people that can help you in your journey to make a positive change?

Name:____

Phone:____________________Email:____________________________

Name:____

Phone:____________________Email:____________________________

Having suicidal thoughts or feeling like you might hurt yourself?

Please go to the nearest emergency room right away. Effective treatment is available to help you through this difficult time.

Understanding your current mood: The Patient Health Questionnaire

This brief questionnaire can help you understand your mood and can also make suggestions on what types of support you might find helpful. Answer the 9 questions to see if this resource is appropriate for you, or if you should contact your health care provider for mood management guidance.

Over the last 2 weeks, how often have you been bothered by any of the following problems?

	Not at all (0)	Several days (1)	More than half the days (2)	Nearly every day (3)
1. Little interest or pleasure doing things	☐	☐	☐	☐
2. Feeling down, depressed, or hopeless	☐	☐	☐	☐
3. Trouble falling/staying asleep, sleeping too much	☐	☐	☐	☐
4. Feeling tired or having little energy	☐	☐	☐	☐
5. Poor appetite or overeating	☐	☐	☐	☐
6. Feeling bad about yourself, or that you are a failure, or have let yourself or your family down	☐	☐	☐	☐
7. Trouble concentrating on things, such as reading the newspaper or watching TV	☐	☐	☐	☐
8. Moving or speaking so slowly that other people could have noticed. Or the opposite; being so fidgety or restless that you have been moving around more than usual	☐	☐	☐	☐
9. Thoughts that you would be better off dead or of hurting yourself in some way	☐	☐	☐	☐

Please add up your scores for each question and write your total here: ______________________________

Guide for Interpreting Scores

Score	Recommended Actions
0-4	At this time, you may not need support for your mood. You can continue using this resource as a support tool.
5-9	Please continue using this resource for helpful information and support.
10-14	Please continue using this resource for helpful information and support. Consider visiting your health care provider for mood management support.
15-19	Please continue using this resource and visit your health care provider for mood management support. You can also refer to the internal and external supports information on page 3 of this resource.
20 or higher	Please visit your health care provider as soon as possible for mood management support. In the meantime, refer to the internal and external supports information on page 3 of this resource.

If you are having suicidal thoughts or feel like you might hurt yourself, please go to the nearest emergency room right away. Effective treatment is available to help you through this difficult time.

Taking control of your health

A new method to quit smoking.

If you feel nervous, angry, worried, sad, or overwhelmed, you may to want to smoke more. One helpful strategy for reducing or quitting smoking is to replace your smoking with pleasant and healthy activities.

How can you do this?

The activities you participate in each day affect your mood. If you increase your number of activities each day that are healthy and make you feel better, you will be more likely to stay quit.

The chart on page 7 can help you track the number of cigarettes you smoke each day, how many pleasant and healthy activities you do each day, and how you are feeling. After recording for a few days or weeks, you might be able to see a connection between your smoking, your activities, and your mood. For example, you might feel better on days when you have more positive activities.

How to use the chart:

Keep this chart where it can be easily accessed, for example, in your wallet.

Every time you participate in a pleasant or healthy activity, or every time you smoke, record it on the chart (please refer to page 6 for examples of pleasant and healthy activities).

- **At the end of the day,** circle in the chart which face best represents how you feel in general:
  - » If you feel content, optimistic, or happy, circle the happy face.
  - » If you feel nervous, worried, or angry, circle the sad face.
  - » If you feel neutral, neither happy or sad, circle the neutral face.
- **At the end of the week,** review the week in this chart and ask yourself:
  1. How many pleasant activities did I participate in each day?
  2. How many cigarettes did I smoke each day?
  3. How did I feel each day?
- Observe how much you have progressed:
  - » Note whether or not your number of pleasant and healthy activities is **increasing**.
  - » Ask yourself: "How can I **increase** my number of pleasant and healthy activities?"
  - » Ask yourself: " Is there any **relationship** between my activity level, mood, and smoking?"

What are pleasant and healthy activities?

Pleasant and healthy activities are activities that make you feel good. They can give you satisfaction and lift your spirits. They can offer you moments of rest, tranquility, and peace or give you energy and joy. They can encourage you to spend more time with family and friends.

Even at work, it is possible to do something pleasant. You may think of someone you love, you may make plans for what you'll do after work, you may remember lyrics to a song, or you can have a nice conversation with friends at lunch.

In addition to helping you feel better, these activities can also help those around you. For example, these activities can help increase the feeling of friendship or family unity, bring you closer together, and increase the chances you will enjoy the time you spend with others.

There are many activities that you can do at no cost but still provide benefits. We provide a list of some of these activities below.

Examples of pleasant and/or healthy activities:

Circle the activities you can see yourself enjoying. If you have tried quitting or reducing smoking in the past, try focusing on activities that you found helpful.

1. Volunteer at a local community centre.
2. Play a game, such as cards, dominoes, etc.
3. Play with your children.
4. Take a walk in the park.
5. Practice relaxation exercises.
6. Take a few deep breaths.
7. Go to a movie.
8. Go out to eat.
9. Spend a happy moment with your family.
10. Hug, kiss your significant other.
11. Put on some music and dance.
12. Go to the beach, the zoo, etc.
13. Read a book of interest to you.
14. Enjoy your favorite TV program, the radio, or music.
15. Play with your dog, cat, etc.
16. Call or visit your parents, children, or friends.
17. Go for a run or play a sport.
18. Finish an important project.
19. Take a moment to pray, meditate, etc.
20. Learn something new.
21. Plan a vacation.
22. Write down some of your happiest moments.
23. Treat yourself to some ice cream.
24. Do a good job on something.
25. Enjoy something beautiful, like a lazy afternoon, a day trip, etc.
26. Join a club.

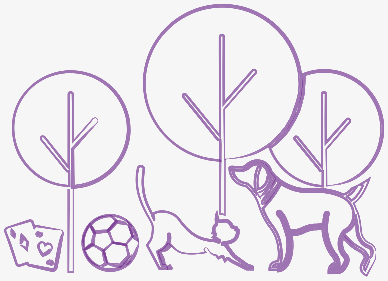

Keeping track

Name:

How many healthy and pleasant activities did you participate in today?

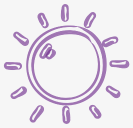

*Do you see a **link** between your pleasant activities and mood?*

DAILY ACTIVITIES WEEK 01 :__/__/__

Day 1	Day 2	Day 3	Day 4	Day 5	Day 6	Day 7
21+	21+	21+	21+	21+	21+	21+
20	20	20	20	20	20	20
19	19	19	19	19	19	19
18	18	18	18	18	18	18
17	17	17	17	17	17	17
16	16	16	16	16	16	16
15	15	15	15	15	15	15
14	14	14	14	14	14	14
13	13	13	13	13	13	13
12	12	12	12	12	12	12
11	11	11	11	11	11	11
10	10	10	10	10	10	10
9	9	9	9	9	9	9
8	8	8	8	8	8	8
7	7	7	7	7	7	7
6	6	6	6	6	6	6
5	5	5	5	5	5	5
4	4	4	4	4	4	4
3	3	3	3	3	3	3
2	2	2	2	2	2	2
1	1	1	1	1	1	1
0	0	0	0	0	0	0

DAILY ACTIVITIES WEEK 02 :__/__/__

Day 1	Day 2	Day 3	Day 4	Day 5	Day 6	Day 7
21+	21+	21+	21+	21+	21+	21+
20	20	20	20	20	20	20
19	19	19	19	19	19	19
18	18	18	18	18	18	18
17	17	17	17	17	17	17
16	16	16	16	16	16	16
15	15	15	15	15	15	15
14	14	14	14	14	14	14
13	13	13	13	13	13	13
12	12	12	12	12	12	12
11	11	11	11	11	11	11
10	10	10	10	10	10	10
9	9	9	9	9	9	9
8	8	8	8	8	8	8
7	7	7	7	7	7	7
6	6	6	6	6	6	6
5	5	5	5	5	5	5
4	4	4	4	4	4	4
3	3	3	3	3	3	3
2	2	2	2	2	2	2
1	1	1	1	1	1	1
0	0	0	0	0	0	0

How many cigarettes did you smoke today?

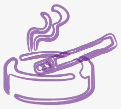

*Do you see a **link** between your smoking and mood?*

DAILY CIGARETTES WEEK 01:

Day 1	Day 2	Day 3	Day 4	Day 5	Day 6	Day 7
21+	21+	21+	21+	21+	21+	21+
20	20	20	20	20	20	20
19	19	19	19	19	19	19
18	18	18	18	18	18	18
17	17	17	17	17	17	17
16	16	16	16	16	16	16
15	15	15	15	15	15	15
14	14	14	14	14	14	14
13	13	13	13	13	13	13
12	12	12	12	12	12	12
11	11	11	11	11	11	11
10	10	10	10	10	10	10
9	9	9	9	9	9	9
8	8	8	8	8	8	8
7	7	7	7	7	7	7
6	6	6	6	6	6	6
5	5	5	5	5	5	5
4	4	4	4	4	4	4
3	3	3	3	3	3	3
2	2	2	2	2	2	2
1	1	1	1	1	1	1
0	0	0	0	0	0	0

DAILY CIGARETTES WEEK 02:

Day 1	Day 2	Day 3	Day 4	Day 5	Day 6	Day 7
21+	21+	21+	21+	21+	21+	21+
20	20	20	20	20	20	20
19	19	19	19	19	19	19
18	18	18	18	18	18	18
17	17	17	17	17	17	17
16	16	16	16	16	16	16
15	15	15	15	15	15	15
14	14	14	14	14	14	14
13	13	13	13	13	13	13
12	12	12	12	12	12	12
11	11	11	11	11	11	11
10	10	10	10	10	10	10
9	9	9	9	9	9	9
8	8	8	8	8	8	8
7	7	7	7	7	7	7
6	6	6	6	6	6	6
5	5	5	5	5	5	5
4	4	4	4	4	4	4
3	3	3	3	3	3	3
2	2	2	2	2	2	2
1	1	1	1	1	1	1
0	0	0	0	0	0	0

How did you feel today?

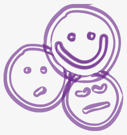

*What **connection** do you see between your mood and your activity today?*

DAILY MOOD WEEK 01:

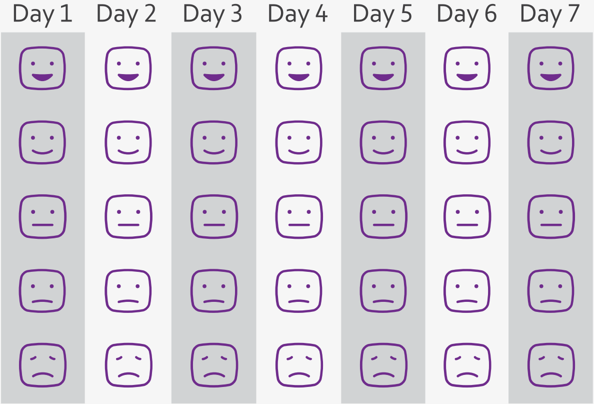

DAILY MOOD WEEK 02:

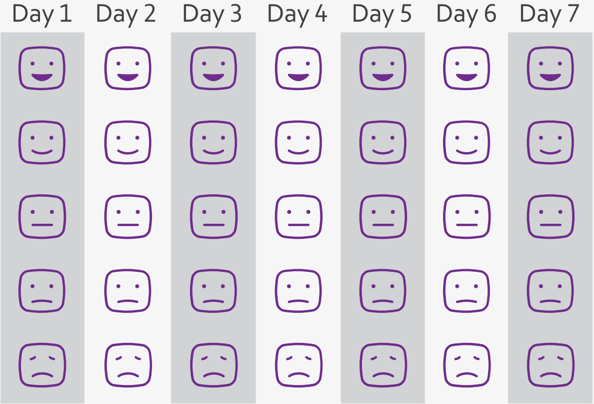

Nonsmoking game plan:

Cravings and urges to smoke are quite common and can be tough to manage. Having a detailed game plan ahead of time can help prepare you for the next time you face a craving or urge to smoke.

Here are some things you might consider when developing your game plan:

- Make it difficult to get to your cigarettes. For example, don't keep cigarettes in your car or at home. Put your cigarettes in the freezer (it's hard to smoke frozen cigarettes!)
- Increase the amount of time you spend in places that do not allow smoking. For example, go for a walk in a mall, or go watch a movie.
- Increase the amount of time doing things that prevent you from smoking. For example, go for a run, or go for a swim at your local community centre.
- Practice how you will tell your friends and family that you prefer they do not smoke in front of you.
- Make use of your support network. For example, ask if you can call a friend or family member when you are feeling down, or feel the urge to smoke.
- Practice saying no when people offer you a cigarette.
- Find activities that make you feel happy. For example, spend time with friends, family or people that make you feel better.
- Reward yourself for your accomplishments. Quitting smoking can be challenging, if you have cut down on the amount of cigarettes you smoke, celebrate this achievement! For example, go for dinner instead of ordering takeout.

These are just a few suggestions. You might have other ideas. Below, list specific strategies for making some changes that will help you become a non-smoker.

Changes to consider	Game plan for change
What will you do to make cigarettes unavailable to you?	
What will you do to increase time spent in nonsmoking places or doing nonsmoking activities?	
Your brother has invited you over for dinner. You know that your brother smokes regularly at home. How will you respond?	
How can you get support from others when quitting?	
What will you say to someone that offers you a cigarette?	
What will you do to manage your mood when you are feeling down?	
What will you do to reward yourself for a job well done?	

Relaxed breathing

When we are anxious or feel threatened, our breathing speeds up in order to prepare our body for potential danger. Relaxed breathing signals the body that it is safe to relax. Relaxed breathing is *slower* and *deeper* than normal breathing, and it happens lower in the body (the belly rather than the chest).

How to do relaxed breathing

To practice make sure you are sitting or lying comfortably; close your eyes if you are comfortable doing so.

Try to breathe through your nose rather than your mouth.

Try to slow your breathing down. Breathe in to a count of 4, pause for a moment, then breathe out to a count of 4.

Make sure that your breaths are *smooth*, *steady*, and *continuous* - not jerky.

Pay particular attention to your out-breath - make sure it is smooth and steady.

In-breath 1...2...3...4 | **Pause** 1... | **Out-breath** 1...2...3...4 | **Pause** 1... | **Repeat**

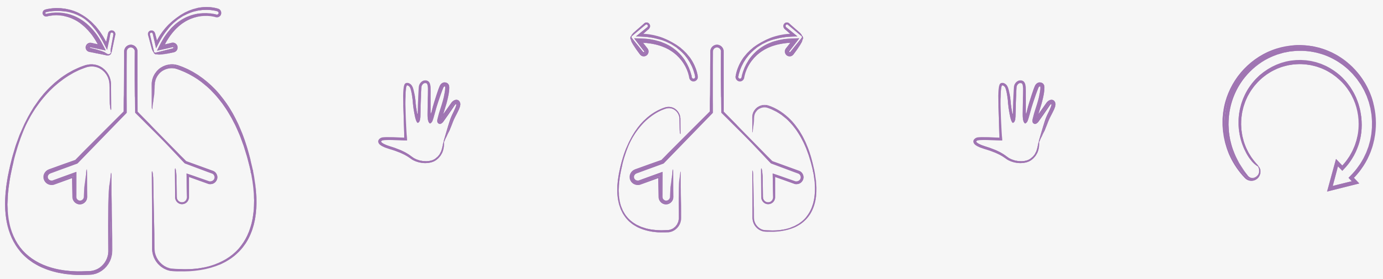

Am I doing it right? What should I be paying attention to?

- Relaxed breathing should be low down in the abdomen (belly), and not high in the chest. You can check this by putting one hand on your stomach and one on your chest.
- Try to keep the top hand still, your breathing should only move the bottom hand.
- Focus your attention on your breath - some people find it helpful to count in their head to begin with *("In ... 2 ... 3 ... 4 ... pause ... Out ... 2 ... 3 ... 4 ... pause ...")*.
- This is a new way of breathing so it can take practice before you get it. Don't give up.

How long and how often?

- Try breathing in a relaxed way for at least a few minutes at a time - it might take a few minutes for you to notice an effect. If you are comfortable, aim for 5-10 minutes.
- Try to practice regularly - perhaps 3 times a day.

Make it work for you

- Find a slow breathing rhythm that is comfortable for you. Counting to 4 isn't an absolute rule. Try 3 or 5. The important thing is that the breathing is slow and steady.
- Some people find the sensation of relaxing to be unusual or uncomfortable at first but this normally passes with practice. Do persist and keep practicing.

Relaxation exercises you can try on your own

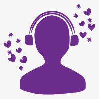

Relaxation exercises, such as meditation, can help you manage feelings of stress and anxiety and improve your overall mood. Below are links to guided relaxation exercises you can try for free online:

Calm provides beginner techniques for mood management. Simply enter your email address and enjoy access to free meditation exercises:

- https://www.calm.com/meditate

The University of Vermont's mindfulness playlist provides a variety of relaxation exercises to help you engage in self-awareness and mindfulness:

- https://soundcloud.com/user-658944755

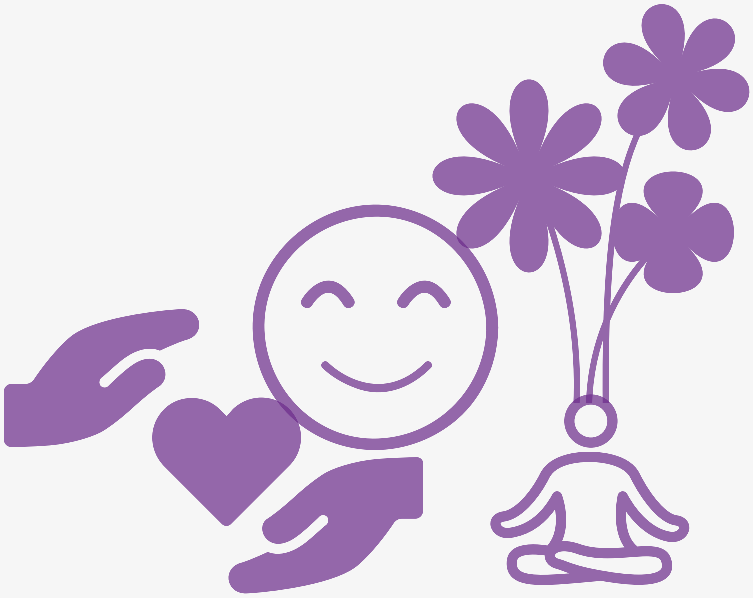

The road ahead

Strategies for maintaining a healthy mood

Depressive episodes do not last forever and your mood will likely improve, even though it might not feel that way when you are depressed or sad. Depressive episodes can occur again in the future – months, years or decades later.

Is there anything you can do to reduce the risk of relapse in mood? Yes. You may or may not be able to eliminate the possibility of having another episode, but you can make episodes less likely, less severe, and less frequent.

Keep up your efforts

When you feel terrible, it's obvious that you need to make your mental health a real priority. When you feel better, it can be tempting to forget all about taking care of yourself. If you feel "good enough", you may want to stop engaging in activities to take control of your health, such as reducing smoking, taking part in healthy activities, and completing relaxation exercises.

Think about the strategies you have been using to cope. Are there some that you will need to keep up over the long term, even after you feel better?

What strategies should I continue to do?

Plan ahead for stress

We all have difficult times in our lives – some of us more than others. For the person who has recently recovered from depression, stressful times may be a risk factor for relapse. The solution is not to avoid all possibility of stress (which none of us can do), but to plan ahead to manage the stress effectively.

Here are some strategies:

When possible, introduce the stress gradually. If, for example, you are returning to work soon, you might check to see if you could go back part-time at first.

Lighten up on ongoing responsibilities. If you are taking a night school course, for example, give yourself permission to eat out more often or have a slightly less tidy home.

Keep up your self-care. How do you keep yourself balanced? Don't give these things up when you need them the most. If a weekly lunch with a close friend is important to you, keep doing it. If you are seeking counseling to help you quit smoking, keep up with your appointments.

Create a mood emergency action plan

If you plan ahead for a relapse, you may be able to get help faster than last time. As a result, the depression may not become as severe, last as long, or be as difficult to recover from. By planning a course of effective action ahead of time, you may not be as anxious, and you may actually reduce the possibility of your depression returning.

If you were to experience a low mood in the future, what are some of the things that you could do to help yourself and get better as quickly as possible?

Here are some areas to think about:

Increase pleasant and healthy activities.
Use examples from the "Taking Control of your Health" exercise.

Don't give up on making positive changes.
Having a low mood can discourage you from pursuing your goals. Hang in there. You will benefit and see an improvement in your mood if you keep trying to reduce or quit smoking, exercise, eat healthy, get enough sleep and get out of the house. Which of these behaviours do you think will help improve your mood the most?

Get support.
Consider giving friends or family members permission to tell you (or perhaps your doctor) when they notice your mood changing.

Take some time to think about how you could get to work early in a depressive episode to prevent it from getting worse. What would have helped this time? Use the list above as a starting point and make up a clear plan of action.

Sources: (1) Abrams et al. (2003). The Tobacco Dependence Treatment Handbook: A guide to Best Practices. New York, NY: The Guilford Press (2) CAMH (2016) Self-awareness alcohol workbook (3) Kroenke et al. (2001). The PHQ-9: Validity of a brief depression severity measure. JGIM, 16, 606-616 (4) Muñoz et al. (1995). Programa Latino para Dejar de Fumar (Latino Program to Stop Smoking). Retrieved from: https://rtips.cancer.gov/rtips/productDownloads.do?programId=105455 (5) Psychology Tools Limited (2017). Relaxed Breathing worksheet. Retrieved from psychologytools.com (6) Simon Fraser University and BC Mental Health & Addiction Services. Antidepressant Skills Workbook. Retrieved from: http://www.comh.ca/publications/resources/asw/SCDPAntidepressantSkills.pdf
