# Supplementary material for: The effectiveness of generic emails versus a remote knowledge broker to integrate mood management into a smoking cessation programme in team-based primary care: a cluster randomised trial
Source: Implement Sci. 2021 Mar 20;16:30. doi: 10.1186/s13012-021-01091-6 (PMC7980670; doi:10.1186/s13012-021-01091-6)
Supplement: Supplementary file 4 — Additional file 4. Sample of generalized monthly email. Description of data: Sample of the first generalized monthly email that was sent to the lead implementer(s) at FHTs allocated to Group B. [file 13012_2021_1091_MOESM4_ESM.pdf]

Greetings STOP Practitioners,

Welcome to the launch of the STOP Program's new quality improvement – Mood Management – initiative! As a STOP practitioner, we are delighted to have you be a part of this exciting change to the STOP Program. As shared in a previous email and recent webinar series: *Integrating a Mood Intervention into Smoking Cessation Programming (part 1 and part 2),* this quality improvement initiative is designed to promote the delivery of brief interventions, and provide access to educational resources, to help you address depression/depressive symptoms with new STOP patients.

Did you know that approximately **13%** of STOP patients report depressive symptoms, which range from minimal symptoms to major depression with severe severity? This can act as a barrier to smoking cessation and increase smoking-related morbidity and mortality. Our goal at STOP is to increase quit success rates among this patient population, by ensuring that treatment for depression is integrated into smoking cessation interventions.

In order to support you with implementing these changes into your practice, we will be sending you emails at the end of each month containing information, current literature and resources we feel will be helpful as you begin to provide brief mood management interventions to your patients.

As part of your first email, we thought it would be useful to share some background literature which supports the integration of mood interventions into smoking cessation programming. A 2013 Cochrane review by van der Meer et al. found that implementing a psychosocial mood management component into standard smoking cessation treatment **increased long-term quit success rates** among patients with current and past depression. For a summary of this Cochrane review and access to the full article please see the resource attached.

We look forward to communicating with you over the coming months! If you have any questions regarding Mood Management at any time, please do not hesitate to contact us at moodinitiative@camh.ca.

Happy Reading!

Mood Management Team

SMOKING CESSATION INTERVENTIONS FOR SMOKERS WITH DEPRESSION

SUMMARY OF COCHRANE REVIEW

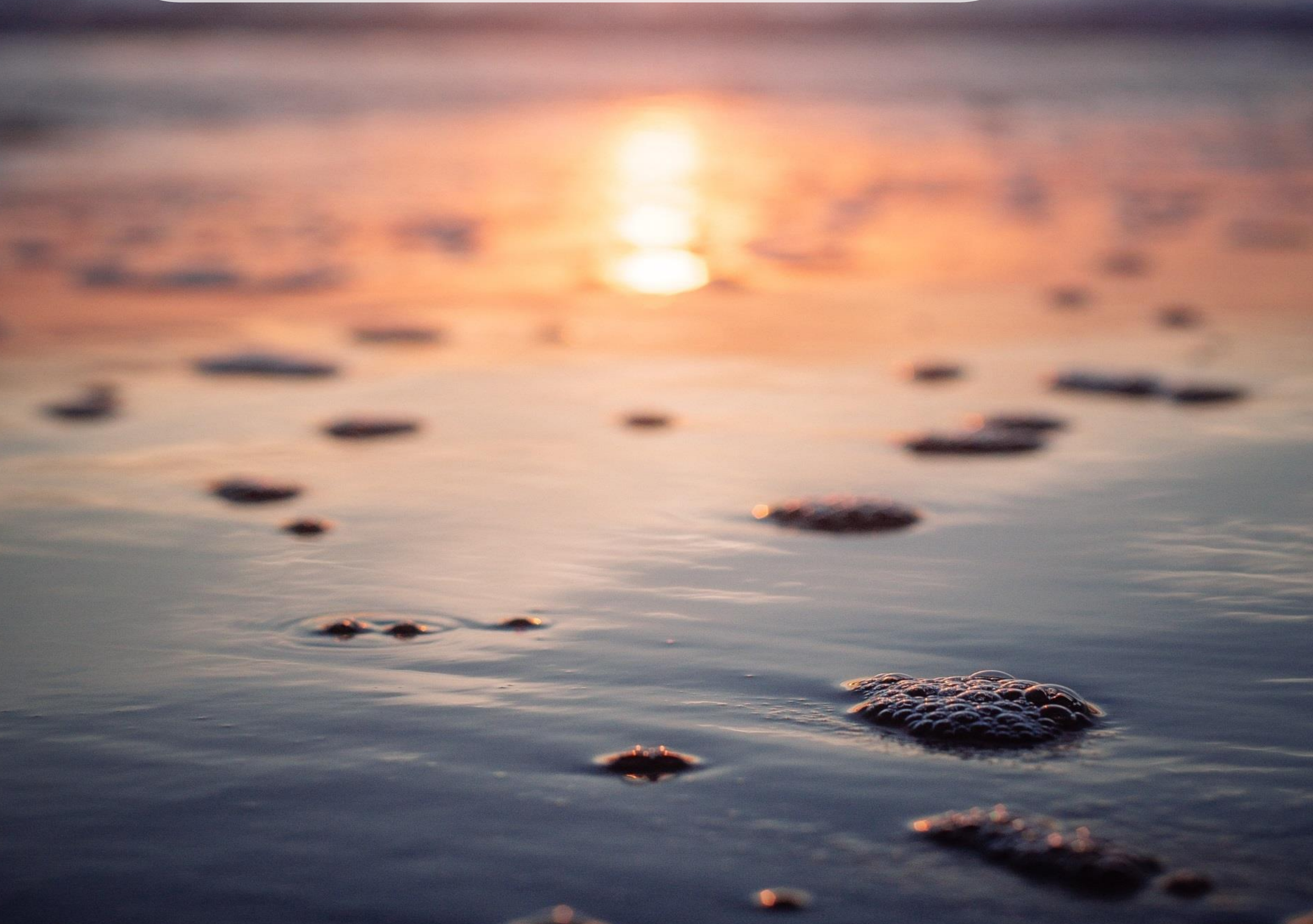

Full text article has been attached to the email.
Abstract can be found here: PubMed link to Abstract (Free)

OBJECTIVE OF THE REVIEW

- Assess the effectiveness of smoking cessation interventions in smokers with current or past depression

BACKGROUND

There is a need for tailored interventions for smokers with depression

- Smoking is strongly associated with depression
- The probability of being a smoker is twice as likely in people with depression [1] [2] [3]
- Smokers attempting to quit are more likely to fail if they are also depressed [4] [5] [6] [7]
- Smokers with depression have a higher chance of:
    - experiencing negative mood changes from nicotine withdrawal,
    - relapsing to smoking after a quit attempt,
    - smoking-related morbidity and mortality [8] [9] [10] [11]
- Several studies have evaluated smoking cessation interventions that involve either antidepressants or psychosocial mood management components [12] [13] [14] [15]

METHODS

- Systematic review which includes randomized controlled trials (RCTs) that compare smoking cessation interventions in smokers with current or past depression
- Comparisons were made between smoking cessation interventions with and without specific mood management components that addressed depression

RESULTS

Adding a brief mood management intervention to smoking cessation treatment helps smokers with current or past depression quit smoking

- Smoking cessation interventions *with* mood management components: (n=33)
    - Addition of mood management has a significant positive effect on smokers with current or past depression as show below: [16]

Figure 1: Forest plot of comparison: Psychosocial mood management versus control for smokers with current depression. Abstinence at six months of greater follow-up.

Study or Subgroup	Experimental Events	Experimental Total	Control Events	Control Total	Weight	Risk Ratio M-H, Fixed, 95% CI
Brown 2007	2	20	6	25	6.6%	0.42 [0.09, 1.85]
Carmody 2008	14	65	6	61	7.7%	2.19 [0.90, 5.33]
Cinciripini 2010	6	33	2	27	2.7%	2.45 [0.54, 11.19]
MacPherson 2010	2	35	0	33	0.6%	4.72 [0.24, 92.85]
Muñoz 1997	5	28	2	25	2.6%	2.23 [0.47, 10.50]
Muñoz 2006a	2	19	1	13	1.5%	1.37 [0.14, 13.57]
Muñoz 2006b	4	20	4	28	4.1%	1.40 [0.40, 4.94]
Muñoz 2009	6	33	4	31	5.1%	1.41 [0.44, 4.52]
Rabius 2007b	23	294	18	249	24.2%	1.08 [0.60, 1.96]
Rabius 2007b	27	256	17	252	21.3%	1.56 [0.87, 2.80]
Van der Meer 2010	29	118	18	119	22.3%	1.62 [0.96, 2.76]
Vickers 2009	1	30	1	30	1.2%	1.00 [0.07, 15.26]
**Total (95% CI)**		**951**		**893**	**100.0%**	**1.47 [1.13, 1.92]**
Total events	121		79			

Heterogeneity: Chi² = 6.12, df = 11 (P = 0.87); I² =)0%

Test for overall effect: Z = 2.84 (P = 0.005)

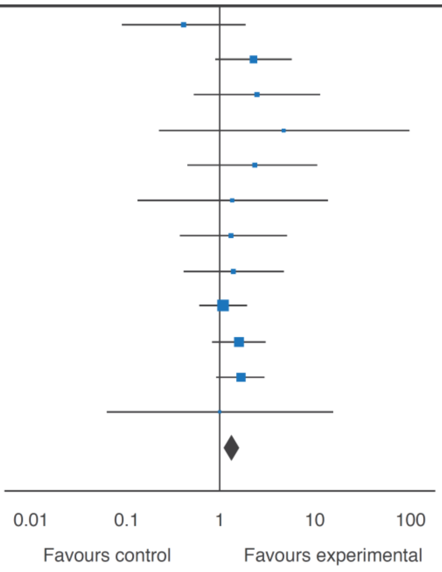

Figure 2: Forest plot of comparison: Psychosocial mood management versus control for smokers with past depression. Abstinence at six months of greater follow-up.

Study or Subgroup	Experimental Events	Experimental Total	Control Events	Control Total	Weight	Risk Ratio M-H, Fixed, 95% CI
Brown 2001	9	86	14	93	12.7%	0.70 [0.32, 1.52]
Brown 2007	5	44	9	64	6.9&	0.81 [0.29, 2.25]
Carmody 2008	12	47	7	55	6.1%	2.01 [0.86, 4.68]
Cinciripini 2010	12	95	8	99	7.4%	1.56 [0.67, 3.65]
Hall 1994	10	29	3	17	3.6%	1.95 [0.62, 6.13]
Hall 1996	5	21	4	23	3.6%	1.37 [0.42, 4.43]
Hall 1998	9	34	5	31	4.9%	1.64 [0.62, 4.37]
Muñoz 1997	10	26	2	27	1.9%	5.19 [1.26, 21.47]
Muñoz 2006a	2	25	6	28	5.3%	0.37 [0.08, 1.68]
Muñoz 2006b	8	25	11	27	10.0%	0.79 [0.38, 1.68]
Muñoz 2009	5	42	4	44	3.7%	1.31 [0.38, 4.55]
Patten 1998	4	13	2	16	1.7%	2.46 [0.53, 11.38]
Van der Meer 2010	58	243	34	242	32.2%	1.70 [1.16, 2.49]
**Total (95% CI)**		**730**		**766**	**100.0%**	**1.41[1.13, 1.77]**
Total events	149		109			

Heterogeneity: Chi² = 15.51, df = 12 (P = 0.21); I² = 23%

Test for overall effect: Z = 3.00 (P = 0.003)

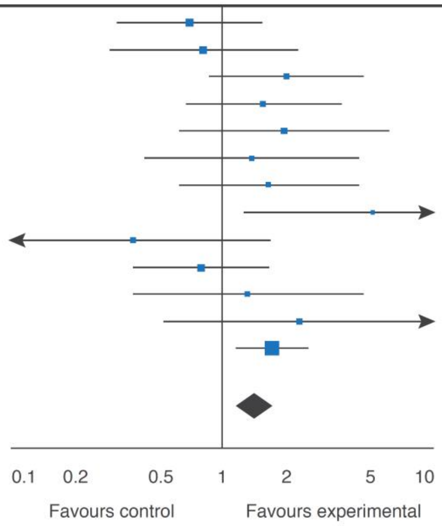

- Bupropion has a positive, but not significant effect on smokers with current depression
- Although evidence is weak due to the small number of trials, bupropion may increase long-term cessation in smokers with past depression [16]

- Evaluation of the effectiveness of fluoxetine, nortriptyline, paroxetine, selegiline, and sertraline in smokers with current or past depression was not possible due to limited trial data [16]

- Smoking cessation interventions ***without*** mood management components: [16]
  - Not enough evidence to show that nicotine replacement therapy or standard smoking cessation interventions are beneficial to smokers with depression [16]

CONCLUSION

- Combining a smoking cessation intervention with a mood management component increases long-term cessation in smokers with current or past depression
- Additional trials are required to evaluate the effectiveness of bupropion and other antidepressants on cessation rates in smokers with current or past depression

BIBLIOGRAPHY

1. *Co-morbidity of smoking in patients with psychiatric and substance use disorder.* **Kalman, D, Baker-Morissette, S and George, TP.** 2005, American Journal on Addiction, Vol. 14, pp. 106-23.

2. *Smoking and mental illness. A population-based prevalence study.* **Lasser, K, et al., et al.** 2000, JAMA, Vol. 284, pp. 2606-9.

3. *Tobacco use and cessation in psychiatric disorders: National institute of mental health.* **Ziedonis, D, et al., et al.** 2008, Nicotine & Tobacco Research, Vol. 10, pp. 1691-715.

4. *Smoking cessation among patients with depression.* **Covey, L.** 1999, Primary Care, Vol. 26, pp. 691-706.

5. *Cigarette smoking: implications for psychiatric illness.* **Glassman, AH.** 1993, The American Journal of Psychiatry, Vol. 150, pp. 546-53.

6. *Past major depression and smoking cessation outcome: a systematic review and meta-analysis update.* **Hitsman, B, et al., et al.** 2012, Addiction, pp. 294-306.

7. *The relationship of major depressive disorder and gender to changes in smoking for current and former smokers: longitudinal evaluation in the US population.* **Weinberger, AH, et al., et al.** 2020, Addiction, Vol. 107, pp. 1847-56.

8. *Quitting smoking does not increase the risk of major depressive episodes among users of Internet smoking cessation interventions.* **Torres, LD, et al., et al.** 2010, Psychological Medicine, Vol. 40, pp. 441-9.

9. *Two decades of smoking cessation treatment research on smokers with depression: 1990-2010.* **Weinberger, AH, et al., et al.** 2013, Nicotine & Tobacco Research, Vol. 15, pp. 1014-31.

10. *Does smoking cessation cause depression and anxiety? Findings from the ATTEMPT Cohort.* **Bolam, B, West, R and Gunnell, D.** 2011, Nicotine & Tobacco Research, Vol. 13, pp. 209-14.

11. *Smoking cessation interventions for patients with depression: A systematic review and meta-analysis.* **Gierisch, JM, et al., et al.** 2012, Journal of General Internal Medicine, Vol. 27, pp. 351-60.

12. *Effectiveness of a mood management component as an adjunct to a telephone counselling smoking cessation intervention for smokers with a past major depression: a pragmatic randomized controlled trial.* **Van der Meer, RM, et al., et al.** 2010, Addiction, Vol. 105, pp. 1991-9.

13. *Effectiveness of cognitive-behavioral therapies for smokers with histories of alcohol dependence and depression.* **Patten, CA, et al., et al.** 1998, Journal of Studies on Alcohol, Vol. 59, pp. 327-35.

14. *Mood management and nicotine gum in smoking treatment: a therapeutic contact and placebo-controlled study.* **Hall, SM, et al., et al.** 1996, Journal of Consulting and Clinical, Vol. 64, pp. 1003-9.

15. *Cognitive-behavioral treatment for depression in smoking cessation.* **Brown, RA, et al., et al.** 3, 2001, Journal of Consulting and Clinical Psychology, Vol. 69, pp. 471-80.

16. *Smoking cessation interventions for smokers with current or past depression (Review).* **van der Meer, RM, et al., et al.** 2013, Cochrane Database of Systematic Reviews, Vol. 8.
